# The Bisphosphonate Accumulation Index (BAI): A Quantitative Metric for Cumulative Antiresorptive Exposure in Pre-Procedural Dental and Surgical Assessment

**DOI:** 10.3390/dj14060364

**Published:** 2026-06-12

**Authors:** Piero Antonio Zecca, Rachele Elisa Miotto, Fabio Brusamolino, Nicolò Vercellini, Marco Serafin, Marina Borgese

**Affiliations:** 1 Department of Medicine and Innovative Technology (DIMIT), University of Insubria, 21100 Varese, Italymarina.borgese@uninsubria.it (M.B.); 2 ASST Settelaghi, Ospedale di Circolo, 21100 Varese, Italy; fabio.brusamolino@asst-settelaghi.it; 3 Lake Como Institute, Academy of Osseointegration, 22100 Como, Italy; nv@vercellini.it; 4 Department of Biomedical, Surgical, and Dental Sciences, Università degli Studi di Milano, 20122 Milan, Italy; marco.serafin@unimi.it

**Keywords:** bisphosphonates, MRONJ, osteonecrosis of the jaw, cumulative dose, risk stratification, pharmacokinetics, antiresorptive therapy

## Abstract

**Background/Objectives**: Medication-related osteonecrosis of the jaw (MRONJ) is a serious complication of bisphosphonate therapy, whose risk is currently assessed through qualitative staging systems that do not integrate pharmacological determinants of cumulative drug exposure. The aim of this study is to present the Bisphosphonate Accumulation Index (BAI), a pharmacologically derived, dimensionless scalar quantifying cumulative exposure to bone-targeted antiresorptive agents by integrating relative potency, administered dose, dosing frequency, route-specific bioavailability, and treatment duration, for use as a pre-procedural assessment tool in patients receiving bisphosphonates. **Methods**: The BAI combines five pharmacologically grounded parameters from peer-reviewed literature: (1) relative antiresorptive potency referenced to etidronate; (2) dose per administration (mg); (3) monthly dosing frequency; (4) bioavailability route; and (5) years of treatment within the preceding 10-year window. The model includes nine bisphosphonates registered in Italy. **Results**: The BAI spans approximately five orders of magnitude (from <1000 for short-term oral therapy to >120,000 for monthly intravenous zoledronic acid). Four analyses support the model: sensitivity analysis identifies relative potency as the main source of variance; ecological calibration against nine MRONJ incidence data points yielded r = 0.911 (*p* = 0.0006, R^2^ = 0.829), indicating that the BAI accounts for approximately 83% of the population-level variance in published incidence rates across heterogeneous regimens (ecological correlation; this does not establish individual-level predictive validity); Monte Carlo simulation on 10,000 patients generated a plausible exposure-strata distribution (6.1% low, 66.6% moderate, 27.3% high); and concordance analysis with a DDD-based metric showed discordance in 7/8 regimens. **Conclusions**: The BAI is a transparent, reproducible, pharmacologically grounded metric of cumulative antiresorptive exposure addressing the quantitative gap identified in the AAOMS 2022 Position Paper. The BAI measures pharmacological exposure, which is a necessary but insufficient component of MRONJ risk; clinical modifiers such as corticosteroid co-administration, diabetes, renal function, and procedure type are not integrated and must be evaluated independently. The provisional exposure strata reported here (<1000, 1000–10,000, >10,000) are hypothesis-generating and intended solely to guide the design of validation studies; they should not be used as clinical decision rules until prospective patient-level validation has been completed.

## 1. Introduction

Medication-related osteonecrosis of the jaw (MRONJ) is a debilitating complication defined by the American Association of Oral and Maxillofacial Surgeons (AAOMS) as the presence of exposed bone or a fistula in the maxillofacial region persisting for more than eight weeks in a patient receiving antiresorptive or antiangiogenic therapy, in the absence of prior local radiotherapy [1]. First reported in 2003 by Marx in patients receiving high-dose intravenous bisphosphonates [2], MRONJ is now recognised across a wide spectrum of antiresorptive agents and clinical contexts, from high-dose oncological regimens to long-term oral therapy for osteoporosis.

The incidence of MRONJ varies widely across therapeutic contexts. In osteoporosis patients receiving oral bisphosphonates, risk estimates range from 0.001% to 0.15% per person-year of exposure [3], whereas in oncological patients receiving intravenous agents, particularly zoledronic acid, the incidence may reach 1–17% [4]. This difference in absolute risk is mechanistically grounded in the pharmacological properties of the bisphosphonate used, particularly its antiresorptive potency, bioavailability, and the cumulative dose delivered to bone.

The pathophysiology is not fully understood, but the alveolar bone of the jaw is unusual: it remodels faster than most skeletal sites and is routinely exposed to mucosal trauma, periodontal disease, and the bacterial flora of the oral cavity. Sustained suppression of osteoclast-mediated remodelling in such tissue is poorly tolerated. Nitrogen-containing bisphosphonates produce that suppression by inhibiting farnesyl pyrophosphate synthase, blocking the mevalonate pathway, and preventing the prenylation of the small GTPases that osteoclasts require to form a functional ruffled border [5]. The result is a tissue that cannot resolve a focal injury such as a tooth extraction, and that may instead progress to exposed necrotic bone. Impaired angiogenesis, soft-tissue toxicity, and altered local immune surveillance contribute to the picture [6,7]. This mechanistic frame motivates the pharmacological focus of the present work: the cumulative drug burden delivered to bone is one of the primary drivers of MRONJ susceptibility, even if it is not the only one.

Despite this pharmacological reality, current clinical guidelines for MRONJ risk stratification remain predominantly qualitative. The AAOMS staging system (Stages 0–3), while widely adopted and clinically useful for disease classification, does not incorporate drug potency, dose, or frequency into its risk assessment framework [1]. The SICMF-SIPMO Italian guidelines similarly offer qualitative risk categories [8]. Serum C-terminal telopeptide (CTX) has been proposed as a pharmacodynamic surrogate for bone remodelling suppression. Still, its clinical utility as an MRONJ predictor is disputed and has not been endorsed by the American Academy of Oral Medicine [9].

Critically, the AAOMS 2022 Position Paper explicitly recognizes this quantitative gap: “the antiresorptive exposure risk for MRONJ may be better defined as a cumulative dose load (e.g., mg equivalent of BP/years of exposure) that would account for risk associated with different medications and dosing schedules over time, similar to the cancer risk associated with tobacco use expressed as pack-years” [1]. To date, no clinically implemented quantitative tool operationalising this concept has been published, although cumulative dose metrics based on defined daily doses (DDDs) have been used in pharmacoepidemiological research [10].

Large-scale epidemiological evidence consistently supports a dose-dependent relationship between bisphosphonate exposure and ONJ risk. A nationwide cohort study of 74,491 women demonstrated that ONJ incidence doubled when the cumulative defined daily dose (DDD) exceeded one year of exposure [10]. Similarly, a retrospective cohort of 152,299 patients receiving intravenous bisphosphonates showed that MRONJ risk was significantly higher when the last dose was administered within 90 days of a dental procedure, with zoledronate requiring a longer drug-free interval than ibandronate for risk reduction [11].

The present paper proposes the Bisphosphonate Accumulation Index (BAI), a pharmacologically derived, dimensionless scalar that quantifies cumulative bone-targeted drug exposure by integrating five pharmacological parameters. The BAI measures pharmacological exposure, a necessary but insufficient component of MRONJ risk. Clinical modifiers such as corticosteroid co-administration, diabetes, renal function, periodontal disease, and the nature of the planned dental procedure are not integrated into the index and must be evaluated independently. The tool is freely implemented as a spreadsheet and designed to be validated prospectively or retrospectively against MRONJ incidence data. This work describes the rationale, construction, pharmacological derivation, and worked clinical examples, and proposes a validation protocol.

The scope of this work is restricted to bisphosphonates, intentionally. Denosumab, the other major antiresorptive associated with MRONJ, does not fit the five-parameter multiplicative model presented here. It is a monoclonal antibody to RANKL, given subcutaneously, with essentially complete bioavailability, a circulating half-life of about four weeks, and no long-term skeletal binding. There is no cumulative bone deposition to integrate. Comparative cohort data nevertheless show that denosumab and high-potency intravenous bisphosphonates produce broadly similar long-term ONJ risk in oncology [12] and osteoporosis [13], which reinforces the clinical value of a quantitative exposure metric even when the drug class differs. Romosozumab and the antiangiogenic agents associated with MRONJ (bevacizumab and the tyrosine-kinase inhibitors) raise the same question in a different form. Each will need its own quantitative framework, and we leave that to future work.

## 2. Materials and Methods

### 2.1. Conceptual Framework

The BAI is constructed on the principle that the osteoclast-suppressive burden delivered to the jawbone is the proximate driver of MRONJ risk. This burden is a function of the amount of pharmacologically active drug that accumulates in the mandibular and maxillary bones per unit time, integrated over the relevant temporal window. Five parameters govern this quantity: the intrinsic potency of the compound, the mass per dose, the dosing frequency, the fraction reaching systemic and skeletal circulation, and the duration of exposure. The BAI integrates these multiplicatively. This formulation assumes linear accumulation of pharmacological burden, which is a first-order approximation and does not account for potential nonlinearities, such as skeletal saturation or remodelling-dependent release dynamics.

The formula for each bisphosphonate product is:

(1) BAI = Relative Potency × Dose (mg) × Monthly Frequency × Bioavailability × Years of Use

When a patient has received multiple bisphosphonate products sequentially or concurrently, the total BAI is computed as the arithmetic sum of the individual product indices. This additive model is conservative. It does not account for competition between bisphosphonate molecules for skeletal binding sites, differences in bone retention kinetics across compounds, or potential non-additive effects on osteoclast suppression in sequential therapy. The additive assumption represents a simplification pending dedicated pharmacokinetic modelling studies.

### 2.2. Relative Antiresorptive Potency

The potency scale embedded in the BAI is referenced to etidronate (assigned value = 1) and extends to zoledronic acid (value = 10,000), reflecting a 10,000-fold range across the class. This scale is derived from the seminal structure–activity relationship studies of Dunford et al. [14], who demonstrated that the ability of nitrogen-containing bisphosphonates (N-BPs) to inhibit human farnesyl pyrophosphate synthase (FPPS), the primary molecular target of N-BPs within osteoclasts, is closely correlated with their in vivo antiresorptive potency (R = 0.74, *p* < 0.0001). The position and configuration of the nitrogen moiety in the bisphosphonate side chain are critical determinants of both initial and final FPPS inhibition constants (Ki and Ki*) [14,15]. This scale reflects relative antiresorptive activity derived from enzymatic inhibition assays and does not represent a direct quantitative predictor of MRONJ incidence; the clinical translation of potency differences is not necessarily linear and is one of the primary reasons why prospective validation of the BAI is required.

Non-nitrogen-containing bisphosphonates (etidronate, clodronate, tiludronate) act through distinct mechanisms (incorporation into non-hydrolyzable ATP analogues and induction of osteoclast apoptosis) and have correspondingly lower potency [16]. The potency hierarchy embedded in the BAI (Table 1) is consistent with established clinical potency rankings and with the FPPS inhibition IC50 data available in the literature [14,17]. The integer values used in the BAI scale (1, 10, 50, 100, 500, 1000, 3000, 5000, 10,000) are conventional clinical approximations on a log-decimal scale that preserve the rank ordering of antiresorptive potency rather than reproducing exact IC50 ratios. These rounded values are consistent with potency rankings reported in the clinical and structure–activity literature [14,15,16] and were selected to keep the BAI calculation transparent and reproducible; the model’s behaviour depends primarily on the relative magnitude of these coefficients rather than on their exact numerical values, an aspect which is explicitly examined by the sensitivity analysis presented in Section 3.2.1.

### 2.3. Dose per Administration

The dose per administration is expressed in milligrams and obtained directly from the approved prescribing information for each product. For products with multiple approved formulations (e.g., alendronate 10 mg daily vs. 70 mg weekly), the clinician enters the prescribed dose. This parameter is not normalised to any reference dose: the BAI deliberately preserves absolute dose information, since it is the mass of drug delivered to bone, rather than the relative convenience of the dosing schedule, that is pharmacologically relevant.

### 2.4. Dosing Frequency (Monthly)

Frequency is expressed as the number of administrations per month (e.g., daily = ~30, weekly = ~4.3, monthly = 1, quarterly = 0.33, annual = 0.083). This approach is more granular than categorical descriptors (“oral” vs. “intravenous”) and captures the practical equivalence between daily and weekly regimens when frequency is already embedded in the formula.

### 2.5. Bioavailability Correction

The single most pharmacologically significant correction in the BAI is the route-specific bioavailability factor. Oral bisphosphonates exhibit extremely low and variable gastrointestinal absorption, ranging from less than 1% to 10% across the class [18,19]. The value of 0.64% (0.0064) used in the BAI reflects the approximate mean bioavailability of commonly used oral bisphosphonates when taken under fasting conditions (alendronate approximately 0.6%, risedronate approximately 0.63%, ibandronate approximately 0.6%), derived from the pharmacokinetic literature [18,19,20]. Although clodronate may achieve a bioavailability of up to 2% [18], and inter-individual variability spans approximately one order of magnitude across the class, a single representative constant is used here as a conservative, operationally tractable approximation. Intravenous formulations, by definition, achieve 100% systemic bioavailability (factor = 1.0). This single correction introduces a 156-fold differential between an oral and an intravenous formulation of the same agent at the same nominal dose. This amplification directly explains the higher MRONJ incidence observed with IV regimens.

Approximately 50% of systemically available bisphosphonate is taken up by bone, with the remainder excreted renally [18]. Since this fraction is constant across the class, it serves as a fixed scaling factor that does not alter the relative BAI values and is therefore absorbed into the potency term’s reference scaling.

A brief note for readers less familiar with bisphosphonate pharmacokinetics may be useful. The 1.0 value for intravenous administration is true by definition: the dose enters the systemic circulation in full. The oral value is the more interesting one. Bisphosphonates are highly polar and calcium-chelating; they cross the intestinal epithelium very poorly and only by the paracellular route, and even that fraction is lost in the presence of food, beverages, or divalent cations. Under standard fasting conditions the absorbed fraction is around 0.6 to 0.7%, with about one order of magnitude of interindividual variability. The single representative value of 0.0064 used in the BAI sits at the centre of this range. The calculator keeps oral bioavailability fixed and does not expose it as a user-adjustable field. Patient-specific bioavailability is essentially never measured in clinical practice, and a free field would invite arbitrary modification that the model is not equipped to absorb. The interindividual variability is instead addressed analytically in the Monte Carlo simulation (Section 3.2.3), where oral bioavailability is sampled from a lognormal distribution; investigators with patient-level absorption data are welcome to modify the spreadsheet directly.

### 2.6. Duration of Exposure: The 10-Year Window

The BAI integrates years of exposure within a 10-year window preceding the assessment date. The pharmacokinetics of skeletal bisphosphonate retention justifies this temporal framing. The terminal half-life of bisphosphonates in bone has been estimated at 10–11 years for alendronate [21], reflecting the rate-limiting step of bone remodelling-dependent drug release. Drugs deposited more than 10 years before assessment contribute a progressively smaller fraction to current bone-remodelling suppression, given that the terminal half-life of bisphosphonates in bone is estimated at 10 years [21]. The 10-year window, therefore, represents an operationally conservative approximation of the pharmacologically relevant exposure period rather than a biologically precise threshold.

This 10-year framing mirrors the established clinical convention in oncological risk metrics (e.g., pack-years for smoking) where the relevant exposure window is defined by the biological half-life of the exposure’s effect, not by the patient’s total lifetime exposure.

One caveat about the 10-year window deserves explicit mention. It assumes physiological rates of bone remodelling. Patients with markedly abnormal bone turnover are the exception. In Paget’s disease, untreated primary hyperparathyroidism, or other high-turnover states, the skeleton releases the previously deposited drug faster, and the effective retention period shortens. The opposite is true when remodelling is profoundly suppressed, as in some patients on prolonged high-dose antiresorptive therapy or in conditions of severely reduced turnover; there, the retention period may lengthen. We retain the 10-year value as the default because no quantitative pharmacokinetic data currently support an individualised adjustment, but clinicians applying the BAI to patients with overt metabolic bone disease should interpret the result with this caveat in mind.

## 3. Results

### 3.1. Worked Clinical Examples

To illustrate the BAI’s discriminative resolution, four representative clinical scenarios are presented (Table 2). These scenarios are hypothetical but are based on approved dosing regimens and typical treatment durations reported in the literature.

• Scenario A (BAI = 4816) represents a postmenopausal woman with osteoporosis receiving weekly alendronate 70 mg for five years. The BAI falls in the moderate-exposure stratum, reflecting the substantial attenuation of systemic bioavailability of oral formulations (factor 0.0064).
• Scenario B (BAI = 9632) represents the same patient after ten years of uninterrupted therapy. The BAI approaches but does not cross the provisional high-exposure boundary of 10,000, consistent with the AAOMS 2022 recommendation to consider a drug holiday specifically at this duration [1]. The progressive increase with duration is consistent with the dose-dependent increase in ONJ risk reported by Park et al. [10].
• Scenario C (BAI = 120,000) represents an oncological patient receiving monthly intravenous zoledronic acid 4 mg for three years. The 25-fold higher BAI relative to Scenario A reflects the combined effect of higher potency (10,000 vs. 500), 100% bioavailability (vs. 0.64%), and the higher absolute dose per infusion, a finding pharmacologically consistent with the dramatically higher MRONJ incidence (1–17%) documented in this population [4].
• Scenario D (BAI = 15,340) illustrates sequential therapy: a patient who received alendronate 70 mg weekly for three years (BAI = 500 × 70 × 4.3 × 0.0064 × 3 = 2890) and then transitioned to annual intravenous zoledronic acid 5 mg (Aclasta schedule for osteoporosis) for three additional years (BAI = 10,000 × 5 × 0.083 × 1 × 3 = 12,450). The total BAI of 15,340 crosses the provisional high-exposure boundary, demonstrating that even a low-frequency intravenous osteoporosis regimen, when following years of oral therapy, places the patient in the high-exposure stratum. The correctly calculated BAI for an annual IV osteoporosis schedule is 8-fold lower than for a monthly oncological schedule (Scenario C), accurately preserving the pharmacological distinction between osteoporosis and oncology regimens.

### 3.2. Computational Validation

In the absence of prospective clinical validation data, four computational analyses were conducted to assess the internal consistency of the BAI model, its ecological agreement with published MRONJ incidence data, its distributional properties in a simulated population, and its discriminative advantage over the existing DDD-based metric. All analyses were performed in Python 3.12 using NumPy, SciPy, and matplotlib.

#### 3.2.1. Sensitivity Analysis

**Figure 1:**
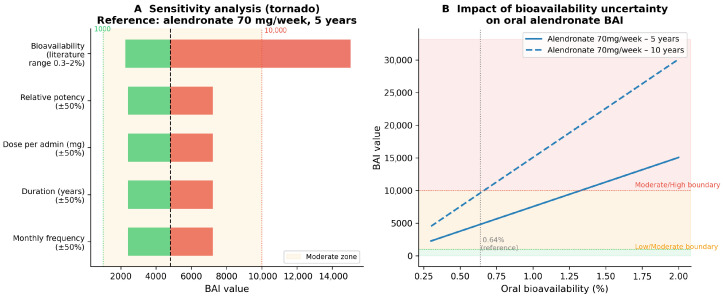
Sensitivity analysis. (**A**): Tornado plot of one-at-a-time parameter variation (reference: alendronate 70 mg weekly, 5 years). (**B**): Impact of oral bioavailability uncertainty (documented range 0.3–2%) on BAI for alendronate over 5 and 10 years.

A one-at-a-time sensitivity analysis was performed on a reference scenario (alendronate 70 mg weekly for 5 years) by varying each of the five BAI parameters within their documented ranges. The results are summarised as a tornado plot (Figure 1A). Relative potency and treatment duration are the parameters that introduce the largest absolute variation in BAI output, followed by dose per administration. Monthly frequency and bioavailability, while clinically important, introduce comparatively smaller uncertainty when held within their published ranges. Panel B of Figure 1 illustrates the specific impact of oral bioavailability uncertainty: across the literature-documented range of 0.3–2.0%, the BAI for 5 years of standard alendronate therapy ranges from approximately 2300 to 15,300, spanning the low-to-moderate exposure boundary but remaining well below the high-exposure boundary. This analysis confirms that the dominant source of BAI uncertainty is the relative potency parameter, which is also the most pharmacologically grounded and least subject to individual variability.

#### 3.2.2. Ecological Calibration Against Published MRONJ Incidence Data

To assess whether the BAI correlates with real-world MRONJ incidence, published incidence data were retrieved from two primary sources: the nationwide Korean cohort study by Park et al. [10], which provides incidence rates stratified by cumulative defined daily dose (DDD) of oral bisphosphonates, and the AAOMS 2022 aggregate data [1], which reports time-stratified incidence rates for oncological patients receiving monthly intravenous zoledronate (4 mg) and intravenous ibandronate (6 mg every 3 months). Each data point was converted to a BAI value using the corresponding published dosing regimen and the formula parameters established in Section 2. This yielded nine independent BAI-incidence coordinate pairs spanning four orders of magnitude in BAI and two in incidence.

**Figure 2:**
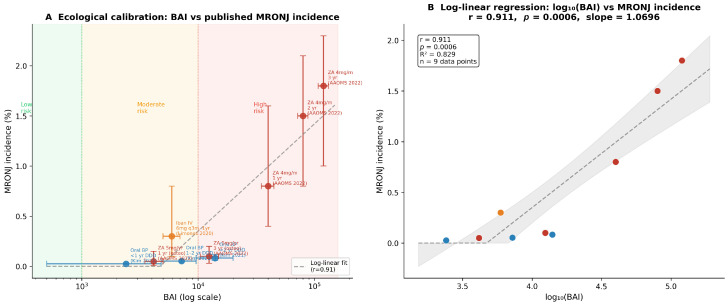
Ecological calibration. (**A**): BAI (log scale) plotted against published MRONJ incidence (%) for nine independent data points derived from Park et al. [10] and AAOMS 2022 [1], with provisional exposure-stratum shading. (**B**): Log-linear regression with 95% confidence band (r = 0.911, *p* = 0.0006, R^2^ = 0.829, n = 9).

Log-linear regression of the nine data points yielded a Pearson correlation coefficient r = 0.911 (*p* = 0.0006, R^2^ = 0.829, Figure 2A,B). The BAI therefore accounts for approximately 83% of the population-level variance in published MRONJ incidence across heterogeneous drug classes, dosing schedules, and patient populations. This ecological correlation should not be interpreted as evidence of individual-level predictive validity: the underlying incidence data are themselves stratified by cumulative defined daily dose, so the BAI and the DDD-based metric share dose and duration as common drivers, and a high R^2^ at the population level does not guarantee that any individual patient with a given BAI will or will not develop MRONJ. The finding supports the pharmacological construct validity of the BAI as an aggregate exposure descriptor; individual-level predictive accuracy remains to be established by prospective patient-level studies. The three provisional exposure-stratum boundaries (BAI = 1000 and BAI = 10,000) are visually consistent with the observed inflexion points in the ecological dose–response curve. All data points from oral bisphosphonate regimens cluster below BAI = 15,000 and below 0.1% incidence, while all IV oncological data points fall above BAI = 40,000 and above 0.8% incidence, with no overlap between the two populations in the BAI space. This ecological separation provides indirect support for the pharmacological rationale of the provisional thresholds.

#### 3.2.3. Monte Carlo Simulation on a Synthetic Cohort

A synthetic cohort of 10,000 patients was generated by sampling from the European distribution of bisphosphonate prescribing, based on published European and Italian prescribing data [22]. The ten most prescribed regimens were included, with weights approximating their relative frequency in the Italian outpatient population. Treatment duration was sampled from lognormal distributions parameterised to approximate real-world treatment durations reported in the literature: median 3–5 years for osteoporosis [23] and approximately 2 years for oncological indications [23]—consistent with published persistence data and drug holiday guidelines. Inter-individual variability in oral bioavailability was modelled as a lognormal distribution (mean 0.64%, sigma 0.4 on the log scale), and oral adherence was sampled from a beta distribution (mean 75%), consistent with medication possession ratios of 79–85% reported for oral bisphosphonate regimens in real-world studies [24,25].

**Figure 3:**
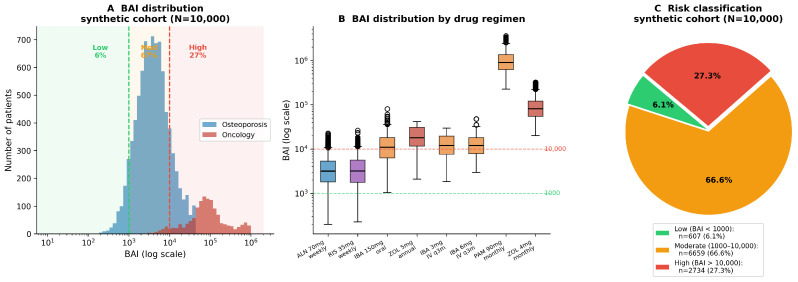
Monte Carlo simulation (N = 10,000). (**A**): BAI distribution histogram by indication (osteoporosis vs. oncology), with exposure-stratum shading. (**B**): BAI distribution by drug regimen (boxplots, log scale). (**C**): Pie chart of exposure-stratum classification.

In the resulting distribution (Figure 3), 6.1% of the synthetic cohort fell into the low-exposure stratum (BAI < 1000), 66.6% into the moderate-exposure stratum (BAI 1000–10,000), and 27.3% into the high-exposure stratum (BAI > 10,000). The predominance of the moderate zone reflects the large proportion of patients on long-term oral alendronate or risedronate therapy in the simulated population. The 27.3% classified as high-exposure closely matches the proportions of oncological and long-duration osteoporosis patients in the distribution, consistent with epidemiological data on antiresorptive prescribing patterns. The boxplot stratified by drug regimen (Figure 3B) confirms that intravenous zoledronate (monthly oncological schedule) and intravenous pamidronate generate BAI distributions entirely within the high-exposure stratum. In contrast, all oral regimens overlap primarily within the moderate-exposure stratum, with tails extending into the high-exposure stratum only at treatment durations exceeding 7 years.

#### 3.2.4. Concordance Analysis: BAI vs. Defined Daily Dose (DDD)

**Figure 4:**
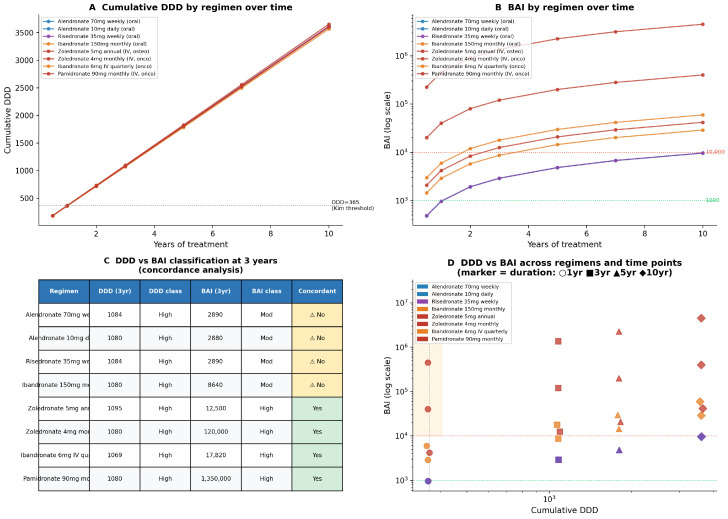
DDD vs. BAI comparison. (**A**): Cumulative DDD trajectories by regimen over 10 years. (**B**): BAI trajectories (log scale) with provisional threshold lines. (**C**): Concordance table at 3 years. (**D**): Scatter plot of cumulative DDD vs. BAI across regimens and time points (markers: ○ 1 yr, ■ 3 yr, ▲ 5 yr, ◆ 10 yr).

The DDD-based metric used by Park et al. [10] represents the current standard for quantifying cumulative bisphosphonate exposure in pharmacoepidemiological research. To evaluate the discriminative advantage of the BAI, a formal concordance analysis was performed across eight representative clinical regimens at the three-year time point; three years was selected as a representative mid-term exposure duration, consistent with the treatment windows recommended for reassessment in major clinical guidelines [1,23] (Table 3 and Figure 4).

Of the eight regimens evaluated, seven showed discordant exposure classification between DDD and BAI at three years; concordance was observed only for monthly intravenous pamidronate (oncology), where both metrics converge on the high category. The remaining seven regimens exhibited systematic discordance in two opposite directions. In four oral regimens (alendronate 70 mg/week, alendronate 10 mg/day, risedronate 35 mg/week, and ibandronate 150 mg/month), cumulative DDD classified the patient as high, whereas the BAI classified the patient as moderate, because DDD does not correct for the ~156-fold lower bioavailability of the oral route. Conversely, in three intravenous regimens (zoledronate 4 mg/month, zoledronate 5 mg/year, and ibandronate 6 mg every 3 months), DDD classified the patient as low or moderate, whereas the BAI classified the patient as high, reflecting the potency and bioavailability terms that DDD does not capture. The DDD system assigns one defined daily dose per standard daily dose regardless of route. Hence, an IV administration and an oral administration of a nominally equivalent dose receive the same DDD count despite a 156-fold difference in bone-delivered drug mass. The BAI corrects for this by incorporating both route-specific bioavailability and drug-specific potency, producing classifications that more accurately reflect the pharmacological exposure reaching the skeleton.

#### 3.2.5. Computational Validation: Summary

**Figure 5:**
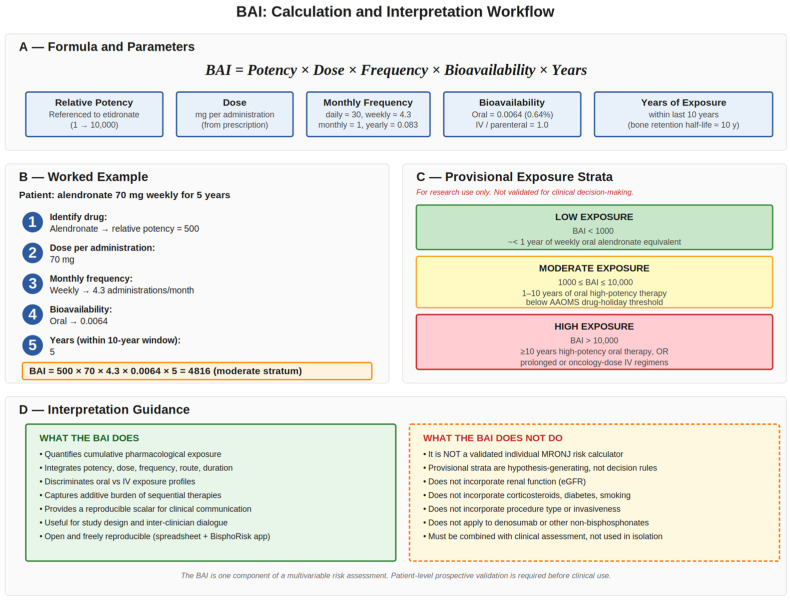
BAI calculation and interpretation workflow. (**A**): Formula and the five pharmacological parameters integrated by the index (relative potency, dose per administration, monthly dosing frequency, route-specific bioavailability, years of exposure within a 10-year window). (**B**): Worked example for a representative osteoporosis regimen (alendronate 70 mg weekly for five years), with step-by-step substitution into the formula yielding BAI = 4816 (moderate exposure stratum). (**C**): Provisional exposure strata with their pharmacological boundaries (low <1000; moderate 1000–10,000; high >10,000), explicitly labelled as research-use-only and not validated for clinical decision-making. (**D**): Interpretation guidance contrasting what the BAI does (cumulative exposure descriptor, oral vs. IV discrimination, sequential-therapy aggregation, reproducible scalar for clinical communication) against what it does not do (it is not a validated individual MRONJ risk calculator; does not incorporate renal function, corticosteroids, diabetes, smoking, procedure type, or non-bisphosphonate antiresorptives; must be combined with multivariable clinical assessment rather than used in isolation). The figure summarises the operational use of the BAI in research settings and is intended as a quick-reference complement to the formal pharmacological derivation in Section 2.

The four computational analyses jointly provide evidence for: (1) the robustness of the BAI to parameter uncertainty within documented biological ranges; (2) ecological agreement with published MRONJ incidence data at the population level (R^2^ = 0.829, *p* = 0.0006), not equivalent to individual-level predictive validity; (3) a plausible exposure distribution when applied to a synthetic European-prescribing-pattern cohort; and (4) a quantifiable difference from the DDD-based metric in seven of eight assessed regimens, attributable to the explicit correction for route-specific bioavailability and drug-specific potency. These findings support the BAI as a pharmacologically coherent exposure metric warranting patient-level prospective validation. A schematic overview of the BAI calculation and interpretation workflow, including the provisional exposure strata and the explicit caveats on clinical use, is provided in Figure 5.

## 4. Discussion

### 4.1. Comparison with Existing Tools

The BAI complements, rather than replaces, the AAOMS 2022 staging system. The staging system classifies established disease; the BAI quantifies pre-procedure pharmacological exposure. Table 4 compares the BAI with currently available risk stratification approaches along key clinical dimensions.

The principal advantage of the BAI over existing approaches is its capacity to generate a continuous, comparable scalar across heterogeneous drug histories. Unlike categorical staging, the BAI can detect small but clinically meaningful differences in exposure, for example, between a patient treated with risedronate 35 mg weekly for 2 years and one treated with alendronate 70 mg weekly for 5 years. These differences are invisible to staging-based systems.

Unlike the CTX threshold proposed by Marx [26], the BAI does not require a blood draw. It is not subject to inter-laboratory variability or to clinical confounders, including corticosteroid use, renal function, and menopausal status, that limit CTX as an MRONJ predictor [26]. The AAOMS itself has moved away from recommending CTX in its 2022 update, citing insufficient evidence [1]. Recent comparative summaries of the major international guidelines (AAOMS, JBMR international consensus, JCO clinical practice guideline) confirm that none of the existing frameworks integrate drug-specific potency, route-specific bioavailability and dosing frequency into a single continuous quantitative descriptor; risk stratification remains primarily qualitative, and drug holidays are recommended on the basis of cumulative duration rather than cumulative pharmacological exposure [27].

A recent quantitative approach using the Jaw-specific Bone Scan Index (ΔBSIJ) has shown promise for early detection of MRONJ in oncological patients [28]. However, this tool requires bone scintigraphy and dedicated software, limiting its use to specialised centres. The BAI, by contrast, requires no imaging, no laboratory testing, and no specialist infrastructure.

Beyond its practical advantages, the BAI makes explicit a pharmacological asymmetry that has important clinical implications. Because oral bisphosphonates exhibit approximately 0.6% gastrointestinal absorption while intravenous administration achieves 100% systemic bioavailability, the route of administration alone introduces a 156-fold difference in bone-delivered drug mass for otherwise comparable nominal doses. This single correction factor dominates the BAI output in most clinical scenarios: ten years of standard weekly oral alendronate produces a BAI of approximately 9600. In comparison, three years of monthly intravenous zoledronate at 4 mg produces a BAI of 120,000, a difference of more than one order of magnitude driven entirely by bioavailability rather than by duration or nominal dose. The BAI therefore reframes the clinical risk question from ‘how long has the patient been taking bisphosphonates’ to ‘how much drug has actually reached the skeleton’, a distinction with direct implications for pre-procedural decision-making. This pharmacological asymmetry is precisely what the available epidemiological data demonstrate: MRONJ incidence is 0.001–0.15% in patients on oral osteoporosis therapy and 1–17% in those receiving intravenous oncological regimens, and the BAI reconstructs this differential mathematically from first principles of pharmacology.

A related implication concerns intravenous osteoporosis regimens, which are perceived as substantially lower-risk than oncological IV protocols. The BAI calculations, however, suggest that even low-frequency intravenous administrations, such as annual zoledronic acid 5 mg for osteoporosis, generate cumulative skeletal exposure values that approach or exceed the provisional high-exposure boundary after 2 to 3 years of treatment. This observation reflects the dominant influence of route-specific bioavailability within the model: complete systemic availability following intravenous administration contrasts sharply with the very limited gastrointestinal absorption of oral bisphosphonates, such that relatively short intravenous regimens may produce cumulative bone exposure levels comparable to or exceeding those observed after many years of oral therapy. This observation should not be interpreted as evidence that osteoporosis IV regimens carry the same clinical risk as oncological schedules; the lower dose per infusion (5 mg vs. 4 mg monthly) and the different biological context mean that absolute risk remains lower. The observation, however, suggests that the cumulative bone-delivered drug mass in this patient group may be higher than intuitively assumed, and that these patients may warrant more careful pre-procedural exposure assessment than current categorical frameworks provide.

### 4.2. Provisional Exposure Strata for Research Purposes: Derivation and Rationale

The boundaries proposed in this section are research-use only. They are not clinical decision rules and should not be used as such until patient-level prospective validation has been completed. Their intended function is to support the design of future validation work, that is, to define exposure ranges that can be used for sample-size estimation and hypothesis testing. Within that framing, a pharmacologically grounded provisional classification is proposed here, anchored on three exposure strata with converging pharmacological and epidemiological rationale (Table 5).

#### 4.2.1. Low-Risk Threshold (BAI < 1000)

The boundary between low and moderate risk is anchored to BAI = 1000, which corresponds to approximately one year of weekly alendronate 70 mg under standard oral bioavailability conditions (500 × 70 × 4.3 × 0.0064 × 1 = 963). This value is not arbitrary: it aligns with the first epidemiologically documented inflexion point in the risk of bisphosphonate-associated ONJ. Park et al., in a nationwide cohort of 74,491 Korean women with osteoporosis, reported a statistically significant and gradual, dose-dependent increase in ONJ incidence (HR 2.36 in higher cumulative DDD categories, *p* < 0.001), consistent with a progressive elevation of risk when cumulative DDD exceeds approximately one year of exposure [10]. The BAI of one year of standard alendronate therapy (≈963) maps to this DDD = 365 boundary. Below BAI = 1000, MRONJ incidence in the oral-therapy population is indistinguishable from background rates reported in non-medicated controls.

#### 4.2.2. High-Risk Threshold (BAI > 10,000)

The boundary between moderate and high risk is set at BAI = 10,000. This value is supported by two independent and convergent lines of reasoning.

First, a pharmacological argument: BAI = 10,000 is the minimum BAI achievable with any approved intravenous high-potency bisphosphonate regimen in an oncological setting within one year of treatment. Even a moderate-intensity oncological IV schedule (ibandronate 6 mg intravenous monthly for one year: 3000 × 6 × 1 × 1 × 1 = 18,000) substantially exceeds this threshold, while no clinically prescribed oral regimen, including the highest-potency oral agent (risedronate 35 mg weekly, the standard approved dose for osteoporosis) at full adherence over three years, reaches it (1000 × 35 × 4.3 × 0.0064 × 3 ≈ 2890). The threshold, therefore, cleanly separates the two pharmacologically distinct populations (oral osteoporosis therapy vs. IV antiresorptive oncological therapy) that define the two ends of the MRONJ incidence spectrum.

Second, a clinical guideline argument: BAI = 10,000 corresponds to approximately ten years of continuous weekly alendronate therapy (500 × 70 × 4.3 × 0.0064 × 10 = 9632). Ten years of high-potency oral bisphosphonate therapy is precisely the duration at which the AAOMS 2022 Position Paper transitions its recommendation from routine monitoring to active consideration of a drug holiday before invasive dental procedures [1]. This alignment is mechanistically coherent: at ten years of bone-targeted accumulation, the pharmacokinetic half-life of the retained drug approaches saturation, and the theoretical residual antiresorptive burden is maximal for oral regimens.

Taken together, BAI = 10,000 defines a threshold above which (a) any patient is either in an IV antiresorptive regimen or has been on oral therapy for a duration universally recognized as high-risk, and (b) the estimated MRONJ incidence based on available epidemiological data transitions from the sub-1% range characteristic of oral therapy to the 1–17% range documented in IV oncological populations [4].

#### 4.2.3. Interpretation and Clinical Use of Provisional Thresholds

These provisional thresholds should be interpreted as a hypothesis to be tested, not as clinical decision rules. Their primary purpose is to guide the design of validation studies, specifically to define the outcome-relevant BAI ranges for power calculations and sample size estimation. A retrospective cohort with BAI values distributed across all three zones, with MRONJ outcomes confirmed by the AAOMS 2022 criteria, would allow empirical ROC analysis to confirm, shift, or reject these boundaries. The authors anticipate that the moderate/high boundary may shift depending on the specific invasive procedure studied (implant surgery vs. simple extraction) and the patient population (osteoporosis vs. oncology).

Clinicians who choose to apply the provisional classification in practice are encouraged to use it as a structured communication tool, namely, to convey quantitative exposure information to specialist colleagues and patients, rather than as a standalone decision algorithm. In all cases, BAI-based classification should be integrated with established risk factors, including corticosteroid co-administration, diabetes, renal function, oral hygiene status, and the nature of the planned dental procedure.

### 4.3. Strengths

The BAI is explicitly framed as a pharmacological exposure metric rather than a standalone clinical risk predictor. This framing is its principal conceptual strength: it makes no claim about MRONJ incidence that is not empirically supported, and it provides a transparent pharmacological foundation for future regression models that will incorporate clinical covariates. Each parameter of the BAI is derived from peer-reviewed pharmacological evidence rather than from empirical calibration on a local clinical dataset. As a consequence, the tool can be applied without prior local validation data, its inputs and calculations are fully reproducible by any clinician or reviewer, and the multiplicative structure correctly captures the interaction between parameters: the same dose of a high-potency IV agent produces a BAI orders of magnitude higher than the same nominal dose of a low-potency oral agent, in agreement with published pharmacological data.

The 10-year window is biologically justified and operationally conservative: it captures the pharmacokinetically relevant retention period while remaining computationally tractable from routine clinical history.

A notable implication of the Monte Carlo simulation is that the low-exposure category represents only a small minority of antiresorptive-treated patients in a realistic European prescribing population. In contrast, the majority occupy an intermediate cumulative exposure zone. This pattern reflects the pharmacological structure of contemporary antiresorptive therapy, in which long-term oral regimens are common and progressively accumulate skeletal exposure over time. In addition, oral patients on high-potency agents for durations exceeding seven years develop BAI distributions with tails extending into the high-exposure stratum, indicating that duration alone can shift an oral-therapy patient from moderate to high exposure without any change in drug or route. The BAI framework, therefore, suggests that many patients receiving treatment for osteoporosis may not be accurately conceptualised as uniformly low-exposure, but rather as occupying a broad intermediate spectrum that gradually shifts toward higher exposure levels with increasing treatment duration. This distributional perspective, which transforms risk from a binary oral-versus-IV dichotomy to a continuous spectrum, may be among the more clinically useful features of a quantitative exposure metric.

### 4.4. Limitations

Several limitations must be acknowledged explicitly. First and most critically, the BAI has not been validated against clinical MRONJ incidence data. No threshold value has been established to separate high-risk from low-risk patients. The tool, therefore, cannot currently be used as a decision rule and should be interpreted as a quantitative exposure summary rather than as a diagnostic test.

Second, the relative potency values are derived from animal studies and in vitro FPPS inhibition assays [14]. The potency term reflects osteoclast-inhibition strength rather than a direct measure of MRONJ risk, and its use as a proxy assumes an indirect biological link that has not been validated against clinical osteonecrosis endpoints. The translation from preclinical potency to clinical MRONJ risk is not linear, and cofactors, including local jaw bone vascularity, oral microbiome composition, corticosteroid co-administration, and genetic susceptibility, are not captured by the BAI.

Third, oral bioavailability is modelled as a single constant (0.64%) despite documented inter-individual variability of up to an order of magnitude [18,19]. This represents one of the major sources of uncertainty at the individual level and may misclassify patients with unusually high or low gastrointestinal absorption.

Fourth, the temporal window is fixed at 10 years as a biologically motivated approximation. Individual patients with markedly abnormal bone turnover (e.g., Paget’s disease, hyperparathyroidism) may retain bisphosphonates for longer or shorter periods.

Fifth, the BAI grows linearly with dose and time by construction. Bisphosphonate accumulation in bone follows saturation kinetics, as skeletal binding sites are finite and remodelling-dependent release creates a time-varying equilibrium. The linear model, therefore, overestimates cumulative exposure in patients on very prolonged regimens compared with a pharmacokinetic compartmental model. This is acceptable for a screening tool but should be considered when interpreting BAI values in patients with treatment durations exceeding ten years.

Sixth, the ecological calibration presented in Section 3.2.2 uses population-level MRONJ incidence estimates rather than patient-level outcome data. Ecological correlations do not necessarily translate to individual-level predictive validity; a high R^2^ at the population level does not guarantee that any individual patient with a given BAI will or will not develop MRONJ. Individual-level predictive validity remains to be established by the prospective validation protocol described in Section 4.5.

Seventh, although the BAI quantifies cumulative pharmacological exposure, MRONJ pathophysiology is mediated by osteoclast suppression and local bone remodelling dynamics. The BAI should therefore be interpreted as an exposure metric rather than a direct measure of biological remodelling suppression, which may vary between individuals due to differences in bone turnover rate, skeletal microenvironment, and systemic comorbidities, including renal function and corticosteroid use. Two patients with identical BAI values may exhibit different degrees of osteoclast inhibition, depending on individual factors.

Eighth, the multiplicative formulation of the BAI (potency × dose × frequency × bioavailability × years) is a pharmacological assumption rather than an empirically derived functional form. This formulation was adopted because pharmacological exposure scales proportionally with dose, administration frequency, and compound potency, consistent with standard pharmacokinetic exposure metrics such as AUC. Alternative formulations, including additive or non-linear models, have not been evaluated and could be explored in future modelling work.

Ninth, the BAI does not incorporate renal function. This is a real omission. Bisphosphonates are cleared unchanged by glomerular filtration, and any reduction in eGFR raises systemic exposure and skeletal accumulation. Preclinical work in chronic kidney disease confirms that skeletal drug levels are higher when renal function is impaired, and that fractionated dosing reduces this accumulation; a recent individual patient-level meta-analysis of randomised trials in CKD shows that the efficacy of bisphosphonate therapy is modified, but not abolished, by impaired renal function [29]. A future version of the BAI could plausibly include an eGFR-based multiplicative correction; defining its functional form requires dedicated pharmacokinetic modelling, which we leave to subsequent work.

Tenth, the BAI is restricted to bisphosphonates. Denosumab and the other non-bisphosphonate antiresorptives sit outside the model by design. Denosumab is a RANKL-targeted monoclonal antibody given subcutaneously, with complete bioavailability, a half-life of about four weeks, and no skeletal binding; there is simply no cumulative bone deposition for a multiplicative integrator to capture. The same point applies, in different form, to romosozumab and to the antiangiogenic agents (bevacizumab, the tyrosine-kinase inhibitors) implicated in MRONJ. Adapting the framework to those drugs is a separate problem and requires a different model structure.

The most important limitation is the most general one. The BAI is a pharmacological exposure metric, not a standalone clinical risk calculator. It does not see the non-pharmacological determinants of MRONJ that any clinician would weigh in practice: corticosteroid co-administration, diabetes, smoking, periodontal disease, oral hygiene, immunosuppression, the type and invasiveness of the planned procedure, and the underlying systemic indication (osteoporosis versus malignancy). All of these matter, and none of them is in the formula. The BAI should be read as the cumulative exposure component of a multivariable judgement, not as a self-contained predictor of individual MRONJ probability. The provisional exposure strata reported in Section 4.2 follow from this logic: they are research-use only, not clinical decision rules, and they remain so until patient-level prospective validation has been completed.

### 4.5. Framework for Future Validation Studies

The BAI is a pharmacologically grounded exposure metric. Whether it carries individual predictive value for MRONJ is a separate question, and one that only patient-level data can answer. The framework below outlines, in broad terms, what such a validation study would look like.

A retrospective cohort design is the most practical first step. Electronic prescribing records linked to dental procedure registries and to outcome ascertainment based on the AAOMS 2022 definition of MRONJ would allow the BAI to be calculated at the moment of the index procedure and outcomes followed over at least 12 months. A prospective design is preferable where the infrastructure permits it, with the BAI computed before the planned invasive procedure. In either case, the covariates that should be collected alongside the BAI are the same: eGFR, corticosteroid use, diabetes, smoking, periodontal status, concomitant antiangiogenic therapy, and the type and invasiveness of the procedure.

The analytical strategy follows naturally. The BAI is best treated as a continuous predictor and characterised by a receiver operating characteristic curve; candidate cut-offs can be identified by the Youden index or by other clinically appropriate criteria. Calibration of observed against predicted incidence across BAI deciles indicates whether the metric tracks the underlying risk. A multivariable model that enters the BAI alongside the clinical covariates listed above will estimate its independent contribution and quantify what it adds beyond the routine clinical workup. As a useful benchmark, recent multivariable machine-learning models that combine cumulative drug exposure with clinical covariates achieve area-under-the-curve values around 0.89 to 0.90 in retrospective MRONJ cohorts [30]; this is the order of magnitude that any BAI-based model should aim to match or exceed.

The provisional strata of Section 4.2 are intended as a starting point for sample-size estimation, not as truth. They are hypotheses to be confirmed, refined, or rejected by the validation study itself. The BAI calculator spreadsheet and the BisphoRisk implementation are released to the research community precisely so that this validation can be undertaken independently and openly.

## 5. Conclusions

The Bisphosphonate Accumulation Index (BAI) is a pharmacologically derived, transparent, and freely implementable metric of cumulative bisphosphonate exposure, intended at the present stage of development as a research instrument and as a structured descriptor for inter-clinician communication, not as a validated standalone clinical decision tool. It addresses the quantitative gap identified in the AAOMS 2022 Position Paper by integrating drug potency, dose, frequency, bioavailability, and duration into a single scalar value spanning five orders of magnitude across the bisphosphonate class. At the population level, the BAI provides pharmacologically grounded discrimination between the exposure profiles of oral osteoporosis regimens and intravenous oncological schedules, and it captures the additive burden of sequential therapies in a transparent way. Whether these population-level differences translate into clinically meaningful individual predictions of MRONJ is a separate question that the present work does not answer and that only patient-level prospective validation can resolve.

Prospective and retrospective patient-level validation studies are required to determine whether the BAI carries individual predictive value for MRONJ, to refine or reject the provisional exposure strata reported here, and to define how it should be combined with the established non-pharmacological risk factors (corticosteroids, diabetes, renal function, periodontal status, procedure type) within a multivariable framework. The provisional strata of less than 1000, 1000 to 10,000, and more than 10,000 are hypothesis-generating and should not be used as clinical decision rules until such validation has been completed. The tool and its underlying spreadsheet are offered to the research community as a starting point for this validation effort. A dedicated mobile application implementing the BAI calculator for all bisphosphonate products registered in Italy is freely available for Android devices (BisphoRisk; https://play.google.com/store/apps/details?id=it.drzecca.bisphorisk (accessed on 29 May 2026)). By combining dose, potency, administration frequency, bioavailability, and treatment duration into a single unified metric, the BAI offers an intuitive analogue to established cumulative exposure concepts such as pack-years in tobacco research. Even before patient-level validation has been completed, this exposure-based approach may facilitate more consistent interpretation of antiresorptive treatment histories and support a more structured dialogue between prescribing physicians and dental clinicians when planning invasive procedures. Exposure-based metrics such as the BAI may represent a step toward more quantitative and reproducible dental risk assessment in patients receiving antiresorptive therapy.

## Figures and Tables

**Table 1:** Bisphosphonate products, pharmacological parameters, and BAI inputs.

Active Principle	Commercial Name (Italy)	Relative Potency	Dose per Administration (mg)	Monthly Frequency (*n*)	Bioavailability	Route
Etidronic acid (disodium salt)	Didronel, Etidros	1	300/200	Variable	0.0064	Oral
Clodronic acid	Clodron, Difosfonal, Bonefos	10	100–400	Variable	0.0064/1 *	Oral/IV
Tiludronic acid	Skelid	50	200	Monthly	0.0064	Oral
Neridronic acid (sodium salt)	Nerixia	100	25–100	0.33 (quarterly parenteral cycle)	1.0	IV/IM
Alendronic acid	Fosamax, Alendros, Adronat	500	10/70	Daily/Weekly	0.0064	Oral
Risedronic acid (sodium salt)	Actonel, Optinate	1000	5/30	Daily/Monthly	0.0064	Oral
Ibandronic acid	Bonviva, Bondronat	3000	150	Monthly/infusion	0.0064/1 *	Oral/IV
Pamidronic acid	Aredia, Pamidrobell	5000	15–90	Infusion	1 *	IV
Zoledronic acid	Zometa, Aclasta	10,000	4/5	Annual/Variable	1 *	IV

* bioavailability set to 1.0. IV = intravenous; Oral bioavailability = 0.0064 (0.64%).

**Table 2:** Worked clinical scenarios demonstrating BAI calculation.

Scenario	Drug	Dose (mg)	Frequency (×/Month)	Years (Last 10)	Index Value
A—Moderate risk (oral, short-term)	Alendronate	70	4.3 (weekly)	5	500 × 70 × 4.3 × 0.0064 × 5 = 4816
B—Moderate/High boundary (oral, 10 years)	Alendronate	70	4.3 (weekly)	10	500 × 70 × 4.3 × 0.0064 × 10 = 9632
C—High risk (IV oncology)	Zoledronic acid	4	1 (monthly)	3	10,000 × 4 × 1 × 1 × 3 = 120,000
D—Sequential therapy	Alendronate + Zoledronic acid (annual)	70/5	4.3/0.083	3 (oral) + 3 (IV) = 6	Alendronate: 500 × 70 × 4.3 × 0.0064 × 3 = 2890; Zoledronate: 10,000 × 5 × 0.083 × 1 × 3 = 12,450; Total = 2890 + 12,450 = 15,340

**Table 3:** DDD class: Low <365, Moderate 365–730, High >730 (annual threshold). BAI class: Low <1000, Moderate 1000–10,000, High >10,000. Concordance is defined as identical class assignment under DDD and BAI; seven of eight regimens are discordant, with concordance observed only for monthly intravenous pamidronate.

Regimen	DDD (3 yr)	DDD Class	BAI (3 yr)	BAI Class	Concordant?
Alendronate 70 mg/week (oral)	1310	High	2890	Mod	No
Alendronate 10 mg/day (oral)	1095	High	2880	Mod	No
Risedronate 35 mg/week (oral)	1309	High	2890	Mod	No
Ibandronate 150 mg/month (oral)	1080	High	8640	Mod	No
Zoledronate 4 mg/month IV (oncology)	360	Low	120,000	High	No
Zoledronate 5 mg/year IV (osteoporosis)	548	Mod	12,450	High	No
Ibandronate 6 mg IV q3m (oncology)	243	Low	17,982	High	No
Pamidronate 90 mg/month IV (oncology)	1080	High	1,350,000	High	Yes

**Table 4:** Comparative features of MRONJ risk stratification tools.

Feature	AAOMS Staging (2022)	SICMF-SIPMO	CTX Threshold (Marx)	BAI (This Tool)
Quantitative output	No (categorical)	No (categorical)	Partial (single biomarker)	Yes (continuous scalar)
Integrates drug potency	No	Partial	No	Yes
Integrates the route of administration	Qualitative only	Qualitative only	No	Yes
Integrates dose and frequency	No	No	No	Yes
Integrates therapy duration	Duration mentioned	Duration mentioned	Implicit	Yes (10-year window)
Applicable pre-procedure	Yes	Yes	Yes	Yes
Validated clinically	Yes	Yes	Disputed	Not yet (proposed)

**Table 5:** Provisional BAI exposure strata with pharmacological and epidemiological rationale. The strata are proposed for research use only (study design, sample-size estimation, hypothesis generation) and are not validated for clinical decision-making at the individual patient level.

Risk Class	BAI Range	Clinical Equivalent	Estimated MRONJ Incidence	Pharmacological Anchor
Low	<1000	<1 year of weekly oral alendronate equivalent	<0.1% (background)	Below Park et al. inflexion point (DDD < 365, HR reference)
Moderate	1000–10,000	1–10 years of oral BP therapy (potency 500–1000)	0.1–1%	Park et al. risk elevation zone; below AAOMS drug-holiday threshold
High	>10,000	≥10 years oral high-potency BP, OR any IV zoledronate/ibandronate	>1%	AAOMS drug-holiday recommendation zone; IV oncology population incidence

## Data Availability

No new data were created or analyzed in this study. Data sharing does not apply to this article. The BAI calculator spreadsheet and the BisphoRisk mobile application (https://play.google.com/store/apps/details?id=it.drzecca.bisphorisk) are freely available.
